# Case Report: A rare case of primary paraganglioma of the gallbladder with a literature review

**DOI:** 10.3389/fonc.2022.1031112

**Published:** 2023-01-26

**Authors:** Yijun Xia, Shi Wang, Xidong Wang, Jiya Du, Lei Zhang, Long Xia

**Affiliations:** ^1^ Department of Hepatobiliary-Pancreatic-Splenic Surgery, Inner Mongolia Autonomous Region People’s Hospital, Hohhot, China; ^2^ Department of Pathology, Inner Mongolia Autonomous Region People’s Hospital, Hohhot, China; ^3^ Department of Pancreatic-Hepatobiliary Surgery, The Sixth Affiliated Hospital, Sun Yat-sen University, Guangzhou, China

**Keywords:** gallbladder paraganglioma, neuroendocrine neoplasms (NENs), immunohistochemistry, differential diagnosis, surufatinib

## Abstract

**Introduction:**

Paragangliomas of the gallbladder are exceptionally rare. To date, only a few cases of this disease have been reported globally, and the majority were found incidentally during surgery. Although complete resection can achieve a curative effect, specific targeted drugs may have survival benefits for patients with potential recurrence and metastasis risks.

**Case presentation:**

A 48-year-old woman was scheduled for anatomical central hepatectomy due to the discovery of a liver mass. Surprisingly, a gallbladder tumor accompanied by intrahepatic invasion was found rather than primary liver lesions during the operation. Postoperatively, the lesion was confirmed to be a paraganglioma originating from the gallbladder with intrahepatic invasion detectable on histopathology. After surgery, the patient was treated with a new targeted drug, surufatinib {200 mg, q.d. [*quaque die* (every day)]}, and no recurrence was observed during the regular follow-up.

**Discussion:**

Gallbladder paraganglioma is rare and occult, and surgeons do not know it well, so it is easily misdiagnosed before surgery. Postoperative pathological examination is the gold standard for diagnosis.

**Conclusion:**

Given that the tumor contained abundant blood sinuses, the early and continuous enhancement of dynamic enhanced CT scanning was its characteristic manifestation. We presented a case in which a primary gallbladder paraganglioma was identified accidentally in a patient who was misdiagnosed with a liver lesion before surgery. Based on our experience in this work, the en bloc resection technique in combination with surufatinib might have a survival benefit to patients at risk of potential recurrence or metastasis; however, further follow-up observations are needed.

## Introduction

In recent years, compared with other solid tumors, the incidence rate of neuroendocrine neoplasms (NENs) has increased significantly. In general terms, NENs can be divided into the following classifications: well-differentiated neuroendocrine tumor (NET), poorly differentiated neuroendocrine carcinoma (NEC), pheochromocytoma (PHEO), and paraganglioma (PARA) (1). Paragangliomas are a special type of NENs called neurogenic NENs, which originate from the neural crest cells of the neuroectoderm. As a whole, they form the so-called paraganglion system, including the adrenal medulla and extra-adrenal neuroendocrine cell groups. Those located in the adrenal medulla are called pheochromocytoma and often produce a large amount of catecholamines (CA), causing a series of clinical symptoms, such as high blood pressure and metabolic alterations. Those located outside the adrenal gland are called paragangliomas, which can further be divided based on whether they arise from the sympathetic or parasympathetic ganglia (2). Paragangliomas originating from glossopharyngeal and vagal nerves distributed along the neck and skull base usually do not produce CAs because they come from the parasympathetic ganglia; instead, those from the sympathetic nervous system are widely distributed from the skull base to the pelvic floor, and have CA secretion functions, which are also called sympathetic paragangliomas (3).

In terms of the symptoms and histological features of “pheochromogenic reaction”, sympathetic paraganglioma is closely related to pheochromocytoma. Given the similarities between sympathetic paraganglioma and pheochromocytoma, the treatment options are essentially the same (3). Paraganglioma can occur in multiple locations on the body, including multiple organs in the head and neck, and the retroperitoneal and urinary systems (4). However, it is worth noting that paraganglioma originating from the gallbladder is extremely rare and has only been reported in a few articles to date, most often after having been discovered incidentally during surgery (5, 6). In this study, we share the case of an interesting and complex patient with preoperatively diagnosed neoplasms in the middle lobe of the liver and an intraoperative finding of a gallbladder mass with a partial intrahepatic invasion rather than a liver tumor. Postoperative pathology confirmed that it was a rare paraganglioma of gallbladder origin. We hope that through a detailed and thorough description of this case, colleagues in hepatobiliary surgery can deepen their understanding of this rare disease.

## Case presentation

**Figure 1:**
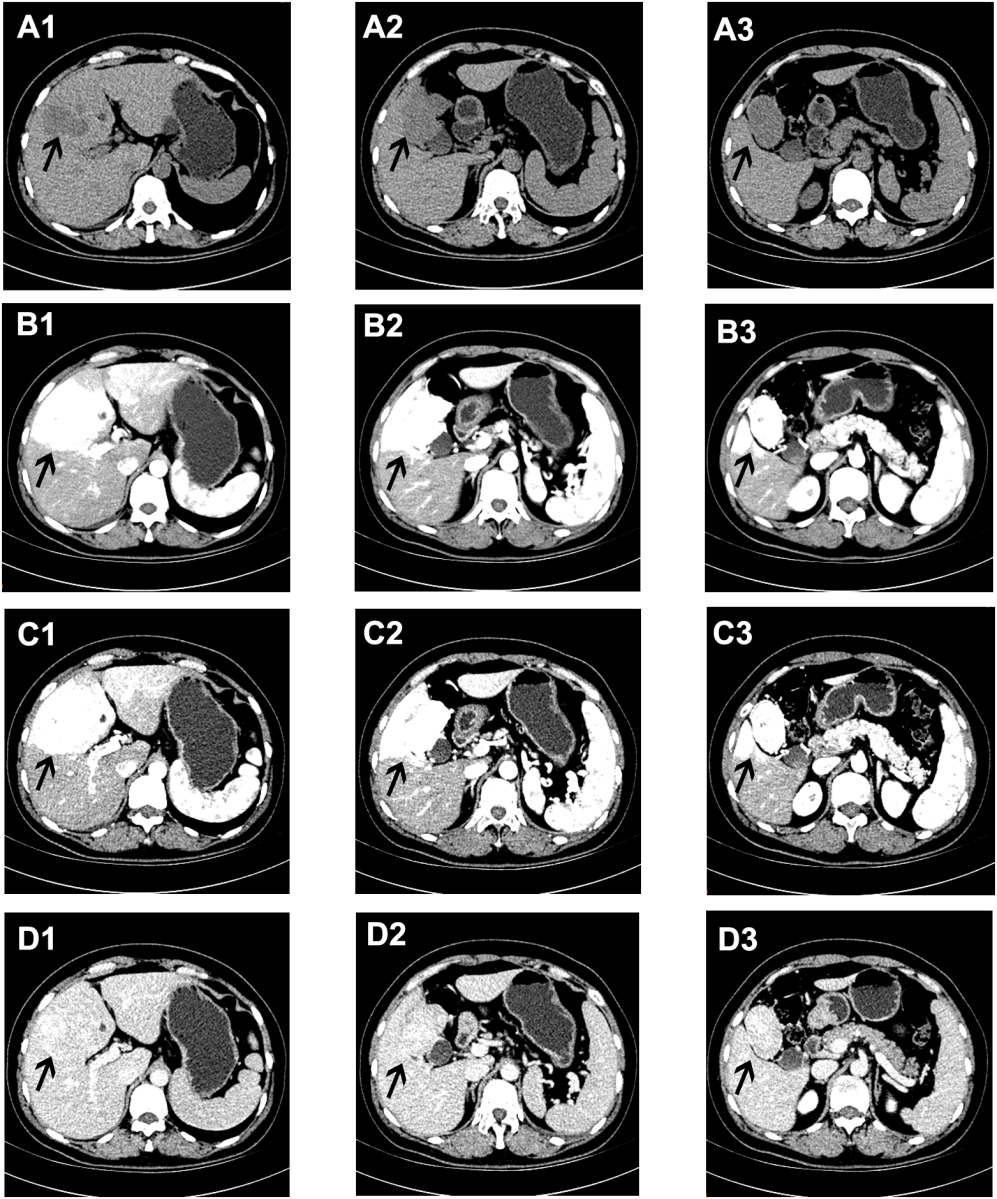
Abdominal enhanced CT manifestations of the lesions in the liver. **(A1–3)** On a plain CT scan, a poorly defined low-density lesion could be seen in the S4b and S5 segments of the liver. In the arterial phase **(B1–3)** and venous phase **(C1–3)** of a contrast-enhanced CT scan, the lesions showed continuous and obvious enhancement, while in the delayed phase **(D1–3)** the enhancement of the lesions decreased, and the density was similar to that of the normal liver, as shown by the black arrow.

**Figure 2:**
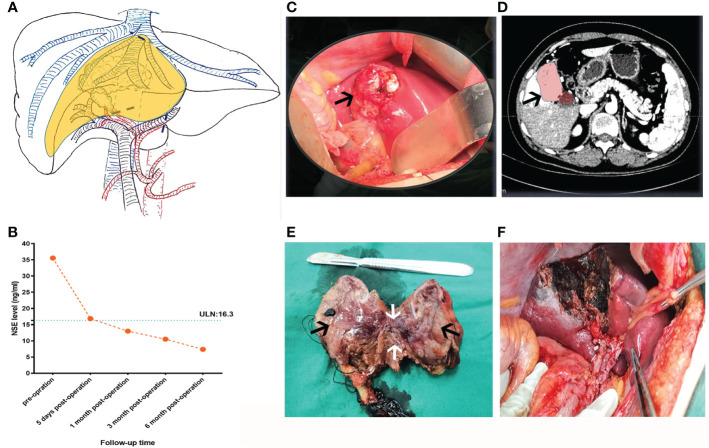
Preoperative planning and intraoperative status of patients. **(A)** According to the information from the two-dimensional enhanced CT image before operation, the hepatic lesion, the operative path **(the black dotted line),** and the resection area **(the yellow area)** were reconstructed by hand-drawing. **(B)** The tendency chart of NSE levels during patient follow-up. **(C)** Intraoperative gallbladder tumors (black arrow) and **(D)** corresponding CT images (black arrow). **(E)** Section of resected gallbladder tumor and part of the liver tissue, as shown, the black arrows indicate the gallbladder mass and the white arrow represents the liver tissue adjacent to the tumor **(F)** The wound condition after operation.

**Figure 3:**
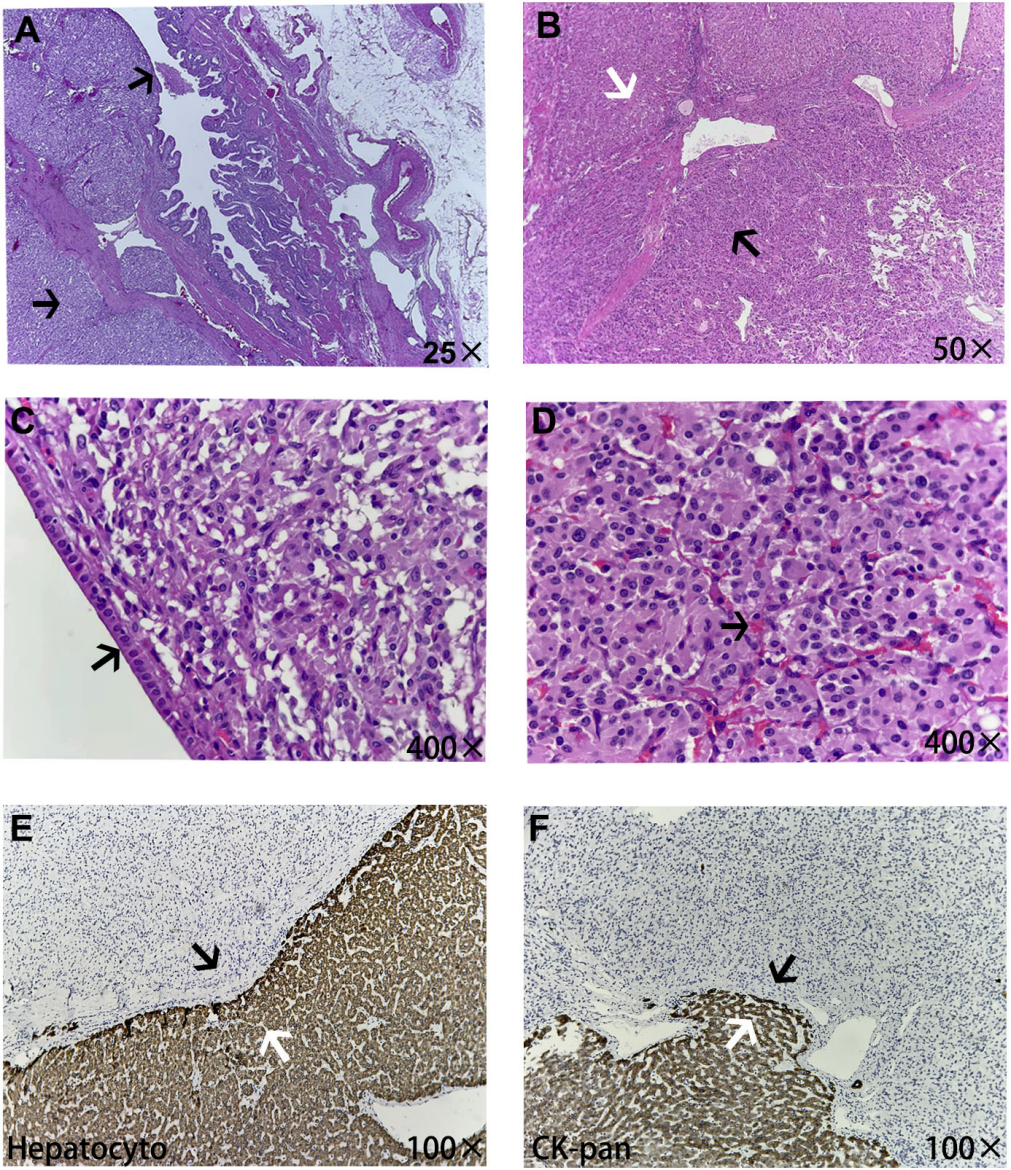
The microscopic pathological structure of tumor tissue. **(A)** The tumor was mainly located in the submucosa of the gallbladder (black arrow). **(B)** The white arrow shows normal liver tissue and the black arrow shows paragangliomas of the gallbladder tissue, in which tumor cells were diffusely distributed in clusters. **(C)** The gallbladder wall, which is surrounded by tumor cells, was covered with simple columnar epithelium structures (black arrow). **(D)** The paraganglioma demonstrated a characteristic zellballen architecture, rich in pink sinusoids inside (black arrow). Parts E and F show the junction structure of the tumor with adjacent liver tissues, and normal liver tissues all expressed the epithelial-derived markers Hepatocyto and CK-pan (white arrows), while non-epithelial paraganglioma tissue was negative (black arrows), which also indicated some degree of tumor invasion of the liver tissue.

**Figure 4:**
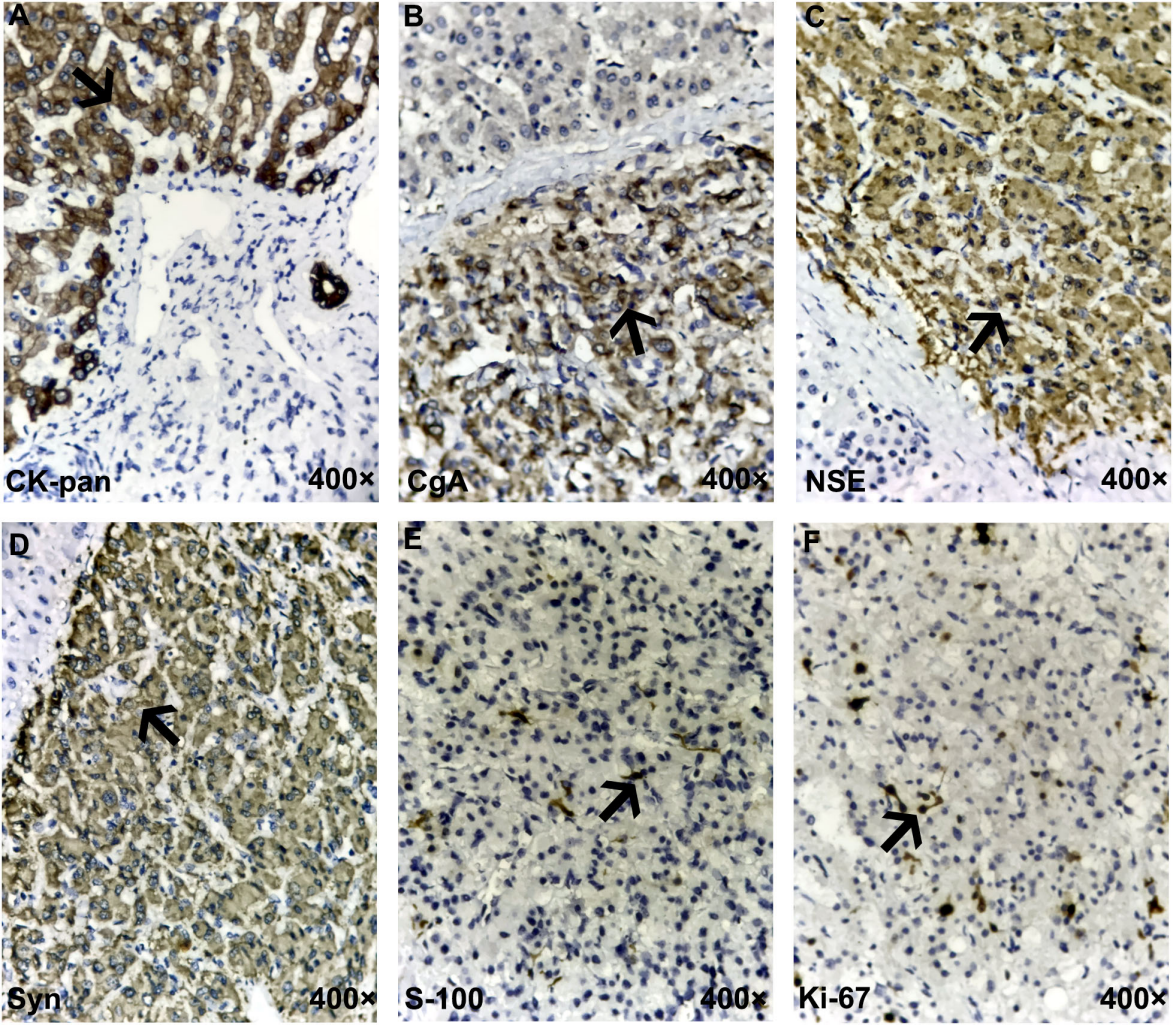
Immunohistochemistry for the gallbladder paraganglioma. **(A)** The difference between neuroendocrine epithelial tumor (NET/NEC) and neuroendocrine non-epithelial tumor (PHEO/PARA) is that the former expresses broad-spectrum pan-cytokeratin (CK-pan), which was negative here, meaning that the tumor was of non-epithelial origin. **(B–D)** Traditional general neuroendocrine markers, including chromogranin A (CgA), neuron-specific enolase (NSE), and synaptophysin (Syn), were all positive. **(E)** S-100 staining highlights surrounding sustentacular cells. **(F)** The Ki-67 proliferation index of tumor cells was 10%. The typical positive expression of each marker in the figures is indicated by black arrows.

A 48-year-old woman was admitted with neoplasms in the middle lobe of the liver, which were diagnosed by abdominal ultrasonography. Abdominal enhanced computed tomography (CT) showed a poorly defined low-density lesion in the middle lobe of the liver (mainly located in the S4b and S5 segments), which was significantly enhanced, and was suspected to be an angiogenic liver tumor (**Figure 1**). Further examination revealed that the levels of neuron-specific enolase (NSE) of the patient were elevated (**Figure 2B**). Combined with her imaging characteristics and our previous clinical experience (7), it was determined that the lesion might be a liver neuroendocrine tumor. The rest of the patient’s examination and laboratory indicators were all normal, and no obvious abnormality was found in the physical examination. Based on the above condition analysis, our preoperative diagnosis was a centrally located liver tumor and that a liver neuroendocrine neoplasm could not be excluded. We planned to perform anatomical central hepatectomy, namely a territory resection around the middle hepatic vein (**Figure 2A**). During the operation we were surprised by the discovery of a gallbladder tumor rather than the liver lesion suggested by CT imaging (**Figure 2C**). Therefore, we quickly adjusted the operative protocol and performed a cholecystectomy, hepatoduodenal ligament lymph node dissection, and partial hepatectomy (**Figures 2E, F**). Meanwhile, the rapid frozen pathological examination during the operation indicated that the margin of the cystic duct and liver was negative. This unexpected intraoperative situation triggered our profound reflection. After the operation, when we reviewed the CT images of the patients again and combined them with the intraoperative situation, we realized that this was an extremely complex gallbladder tumor. The bottom and body of the gallbladder had been filled with solid masses and enhanced significantly, whereas the density of the neck of the gallbladder was still normal (**Figure 2D**). As for the clear enhancement of the liver around the gallbladder (i.e., what we had mistaken for liver lesions before surgery), this was most likely related to abnormal perfusion or the existence of an arteriovenous fistula. Postoperative pathological analysis revealed that the size of gallbladder lesion was about 6.0 cm × 4.0 cm × 3.5 cm, which invaded the whole layer of gallbladder wall and partly involved the adjacent liver tissue. Combined with immunohistochemical staining, it was consistent with paraganglioma of gallbladder. Microscopically, the lesion was mainly located within the submucosa of the gallbladder. The tumor cells were diffusely distributed in clusters, mainly composed of principal cells and sustentacular cells. The chief cells were mostly arranged in nests, forming a characteristic “zellballen” architecture, and the cell clusters were filled with blood sinuses (**Figure 3**). Further detailed immunohistochemical analysis revealed that broad-spectrum pan-cytokeratin (CK-pan) antibody staining was negative, which means that the tumor originated from non-epithelial tissue. Chromogranin A (CgA), neuron-specific enolase (NSE), and synaptophysin (Syn), three important neuroendocrine markers, were all positive; S-100 highlighted surrounding sustentacular cells, and the Ki-67 proliferation index of tumor cells was 10% (**Figure 4**). To further exclude metastatic paraganglioma, the patient underwent a post-operative systemic positron emission tomography-computed tomography [specifically ^18^F-FDG (fluorodeoxyglucose F 18) PET-CT] scanning, which revealed no other hypermetabolic lesions. Collectively, the final diagnosis was primary gallbladder paraganglioma with partial liver invasion. In view of the abundant blood supply of the tumor and its potential for malignancy, we recommended that the patient take surufatinib, a new oral tyrosine kinase inhibitor developed completely independently in China at 1 month after surgery. Surufatinib has dual anti-angiogenic and immunomodulatory activity, and has been shown to be effective in clinical trials in non-pancreatic neuroendocrine tumor patients. We regularly followed up with the patient for half a year; her general condition was fine. Serum NSE levels showed a continuous downward trend (**Figure 2B**), and no obvious abnormality was found in biochemical tests and imaging examinations.

## Discussion

Paragangliomas are neoplasms originating from the paraganglia, which develop from neural crest cells and form the scattered nerve endocrine system around or within the autonomic nervous system, which extends from the skull base to the pelvic floor. In general, paragangliomas in the adrenal medulla are called pheochromocytoma, while those outside the adrenal gland are designated extra-adrenal paragangliomas, also known as neurogenic NENs (2). Extra-adrenal paragangliomas are common in the body of the carotid artery or the bulb of the jugular vein, and, occasionally, occur in the bladder, prostate, and cauda equina; primary gallbladder paragangliomas are very rare in clinical practice (6). Theoretically, primary gallbladder paragangliomas arise from the migration of the hepatic plexus paraganglioma, which innervates the gallbladder, and is formed by sympathetic and parasympathetic fibers of the left vagus and celiac plexus (8).

At present, we still know very little about primary gallbladder paragangliomas. Since the first report of a gallbladder paraganglioma by Miller et al. in 1972 (9), the literature on gallbladder paraganglioma has been very limited, consisting entirely of case reports (**Table 1**) (5–7, 10–19). To our knowledge, our case is the largest tumor in all the current reports of the disease, and the pathology showed that it was accompanied by adjacent liver invasion, showing a certain degree of aggressiveness. Owing to the lack of typical clinical symptoms, some of these cases were found accidentally during cholecystectomy, and the others were due to the occurrence of accompanying symptoms or complications, such as right upper abdominal colic, obstructive jaundice, and bleeding. By reviewing these case reports, combined with our experience, we found that some of the clinical characteristics of primary gallbladder paraganglioma are as follows: (1) the patients were predominantly middle-aged women; (2) most patients lacked typical clinical symptoms and signs; (3) the CT scan of the lesion usually showed a soft tissue density mass with a clear boundary in the gallbladder, a small volume, and no obvious thickening and invasion of the surrounding gallbladder wall; (4) because the tumor contained abundant blood sinuses, most of them showed obvious enhancement in the early phase, the degree of enhancement could be close to the blood pool, the portal phase continued to enhance, and the delayed phase subsided; (5) the patients’ serum NSE level may be elevated (at least in this case study); and (6) most of the tumors were located in the subserous layer of the gallbladder. It should be pointed out that these lesions still need to be differentiated from gallbladder polyps and mass-type gallbladder carcinoma. Gallbladder polyps are small and frequent, and the maximum diameter of the lesions is more than 1cm. Mass-type gallbladder carcinoma could also be manifested as a heterogeneous hyperenhancing soft-tissue mass, but often accompanied by necrosis, resulting in morphological changes of the gallbladder and the thickening or invasion of the gallbladder wall, but its degree of enhancement is significantly lower than that of paraganglioma. Although anatomical imaging for the precise localization of pheochromocytomas/paragangliomas (PGLs) is crucial, functional imaging is becoming an integral part of tumor imaging, especially for patients with multiple metastases. It was expected that patients with PHEO/PGL will benefit from the evaluation of functional characteristics of these tumors and new image-based treatment schemes (20, 21).

**Table 1:** Summary of cases with paraganglioma of the gallbladder.

Case no.	Source year	Age (years)/sex	Clinical manifestation	Imaging characteristics		Size (cm)	Location	Associated findings	Hormone secretion
**1**	Miller (9), 1972	67/M	Recurrent hematemesis	Duodenal ulcer next to scarred duodenal bulb		3.0	NM	Tumor bleeding cholecystoduodenal fistula	NM
**2**	Wolff (10), 1973	32/F 52/F 59/F	Cholelithiasis Cholelithiasis Cholelithiasis	NM NM NM		NM NM NM	Subserosal Subserosal Subserosal	Chronic cholecystitis Chronic cholecystitis Chronic cholecystitis	NM NM NM
**3**	Kawabata (11), 1999	51/M 55/M	Hypochondralgia Epigastralgia	Gallbladder stones Hepatobiliary lithiasis		0.5 × 0.3 0.3 × 0.2	Subserosal Subserosal	Liver dysfunction Chronic cholecystitis	NM
**4**	Hirano (12), 2000	58/F	Right hypochondrial pain	Lesion in the neck of gallbladder		1.3 × 0.9	Submucosal	None	No
**5**	Cho et al. (13), 2001	45/F	Intermittent right upper quadrant pain	Fundal mass with diffusely thickened wall		2.5	NM	Tumor bleeding with chronic cholecystitis	No
**6**	Mehra (5), 2005	36/M	None	Normal		1.5	Subserosal	Chronic cholecystitis	NM
**7**	Sakuma (14), 2011	38/M	Hepatic hilus tumor	Heterogeneous mass and cholelithiasis		NM	Subserosal	Ectopic pheochromocytoma	NM
**8**	İlhan Ece (15), 2015	57/F	Intermittent right upper quadrant pain	Gallbladder stones and a mass within the triangle of Calot		1.8	Subserosal	Chronic cholecystitis	NM
**9**	Raha (16), 2018	NM	Right upper quadrant pain	Gallbladder stones		NM	Subserosal	Paraganglioma of the cystic duct	No
**10**	Karel (17), 2019	36/M	Mild hypertension and tinnitus	A lesion with arterial enhancement adjacent to the gallbladder		2.2	NM	Elevated plasma dopamine level associated with SDHD mutation	Yes
**11**	Furrukh (6), 2020	63/F	Recurrent biliary colic	Mildly dilated gallbladder		NM	Submucosal	Chronic cholecystitis	No
**12**	Shukla (18), 2021	72/F	Discomfort in both ears accompanied by hearing loss	A lesion was seen in the neck of the gallbladder		2.3 × 2.2	NM	Familial paraganglioma syndromes	NM
**13**	Present case	48/F	Physical examination revealed a liver mass	A tumor with a rich blood supply in the middle lobe of the liver		6.0 × 4.0	Submucosal	Paraganglioma of the gallbladder	No

*NM indicates not mentioned.

A thorough and detailed pathological examination after the operation was critically important for the diagnosis and differential diagnosis of NENs. The difference between NETs/NECs (neuroendocrine epithelial tumors) and PHEO/PARA (neuroendocrine non-epithelial tumors) is that the former has keratin expression, which was negative for spectral keratin in our patient (1). Microscopically, paraganglioma has its characteristic structure, which has the appearance of organoid “zellballen” architecture. The major and most common cell types are oval and granular chief cells, which are usually positive for CgA, NSE, and SYN, and negative for cytokeratin. Another cell-type component, which is usually less easily recognized in Hematoxylin eosin (HE), is a supportive sustentacular cell; usually presented as a fusiform hyperchromatic nucleus and an ambiguous vacuolar cytoplasm, it can be detected by positive immunostaining for S-100 (22). To sum up, pathological examination is the gold standard for the diagnosis of paraganglioma. Its characteristic immunohistochemical profile (i.e., positive for SYN, CgA, NSE, and S-100, and negative for cytokeratins) is helpful for the definite diagnosis and avoiding potential diagnostic pitfalls.

Currently, it is commonly believed that about 35%–40% of paragangliomas/pheochromocytomas (PPGLs) are genetically related (8). Therefore, patients with a definite diagnosis of PPGLs need to undergo pathogenic gene testing to determine whether or not their PPGLs are hereditary. In the case we reported, the patient did not have symptoms such as hypertension and palpitation. On detailed inquiry, there was no history of a similar condition in any family member. Because our hospital did not yet have the resources for genetic testing, and because of the high levels of COVID-19, the patient was unfortunately unable to undergo genetic testing. Since the fourth edition of the WHO classification, paragangliomas are no longer classified as benign and malignant because both may have metastatic potential and there are no well-defined features that can effectively predict metastatic behavior. This view was maintained in the fifth edition of the WHO bluebooks, which was published in 2022. Before recognizing that all PPGLs have variable metastatic potential, academics tried to develop multiple scoring or classification systems to predict metastatic risk. Although these assessment models have some predictive value, they lack universality and practicality. Therefore, the 2022 WHO bluebook did not highly recommend the metastatic risk assessment system of PPGLs (23).

In light of the fact that extra-adrenal tumors are more commonly clinically aggressive than adrenal tumors, and that in this case the tumor partially invaded the liver, although we had implemented a surgical scheme similar to the radical resection of a gallbladder carcinoma, postoperative recurrence problem is always a concern. As a new type of oral tyrosine kinase inhibitor, independently developed in China, surufatinib (24) has the dual activities of anti-angiogenesis and immune regulation. Encouragingly, it has shown good results in a Phase III clinical trial in China for patients with advanced non-pancreatic neuroendocrine tumors, solving the problem of patients with non-pancreatic neuroendocrine tumors lacking effective drug treatments (25, 26). Therefore, at 1 month after surgery, we recommend that the patient takes oral surufatinib to prevent the adverse consequences of recurrence and metastasis. So far, the patient from our case study has been followed up regularly for more than 6 months, and was in good condition at time of writing, with no signs of metastasis and recurrence.

## Conclusion

In conclusion, primary paragangliomas of the gallbladder are extremely rare in clinical settings and little is known about them. High levels of vigilance towards preoperative imaging is required for the detection of lesions that show significant and persistent enhancement early on the enhanced CT within the gallbladder. The characteristic immunohistochemical expression profile and the detection of pathogenic genes are critically important for diagnosis. In the era of targeted therapy, surufatinib might be a more effective treatment option for such tumors that have a risk of metastasis and recurrence, although more high-grade, high-quality clinical studies are needed to prove this in the future. We hope that through this case, more doctors can deepen their understanding of this rare disease and learn from our experiences and lessons.

## Data availability statement

The raw data supporting the conclusions of this article will be made available by the authors, without undue reservation.

## Ethics statement

The studies involving human participants were reviewed and approved by Ethics Review Board of the Inner Mongolia People’s Hospital. The patients/participants provided their written informed consent to participate in this study. Written informed consent was obtained from the individual(s) for the publication of any potentially identifiable images or data included in this article.

## Author contributions

YX, SW, and LX collected imaging data and wrote the article, JD performed histopathological analysis of tissue sections, and LZ and LX reviewed and revised the manuscript. All authors contributed to the article and approved the submitted version.
